# Prognostic Significance of Ultrasound Findings of Acute Acalculous Cholecystitis for Elderly Long-Term Bedridden Patients

**DOI:** 10.3389/fmed.2021.743998

**Published:** 2021-10-08

**Authors:** Qian Lin, Lei Shen, Cheng Chen, Zhen Yang, Yifan Que, Yani Liu, Ming Yin, Guogang Xu, Junlai Li

**Affiliations:** ^1^Department of Medical Ultrasonics, The Second Medical Center & National Clinical Research Center for Geriatric Diseases, Chinese PLA General Hospital, Medical School of Chinese PLA, Beijing, China; ^2^Department of Gastroenterology, Faculty of Digestive Medicine, The First Medical Center, Chinese PLA General Hospital, Medical School of Chinese PLA, Beijing, China; ^3^The Second Medical Center & National Clinical Research Center for Geriatric Diseases, Chinese PLA General Hospital, Medical School of Chinese PLA, Beijing, China; ^4^Department of Radiology, The First Medical Center, Chinese PLA General Hospital, Medical School of Chinese PLA, Beijing, China; ^5^Department of Geriatric Medicine, The Second Medical Center & National Clinical Research Center for Geriatric Diseases, Chinese PLA General Hospital, Medical School of Chinese PLA, Beijing, China; ^6^Department of Medical Ultrasound, Tongji Hospital, Tongji Medical College, Huazhong University of Science and Technology, Wuhan, China; ^7^Department of Emergency, The Second Medical Center & National Clinical Research Center for Geriatric Diseases, Chinese PLA General Hospital, Beijing, China

**Keywords:** acute acalculous cholecystitis, elderly, gallbladder, long-term bedridden patients, ultrasound

## Abstract

**Background:** Acute acalculous cholecystitis (AAC) is characterized by the development of cholecystitis in the gallbladder without gallstones or with small gallstones unrelated to inflammatory diseases. This disease is not rare in the elderly bedridden patients with co-morbidities and prone to develop life-threatening gangrene or perforation of gallbladder. Early imaging is essential for detecting and effectively treating AAC. This study aimed to evaluate the use of ultrasound diagnostic criteria for the diagnosis and prognosis of elderly long-term bedridden patients with suspected AAC.

**Methods:** We retrospectively studied 374 elderly bedridden patients with clinical manifestations of AC at the acute stage of the disease. Gallbladder anomalies were found in 92 patients by ultrasound examination, which correlated with the duration time of clinical manifestations, complications, as well as therapeutic prognosis. The major and minor ultrasound criteria of AAC were made according to the Tokyo Guidelines 2018. Ultrasound results were thought to be AAC positive when they met two major criteria or one major and two minor criteria.

**Results:** Forty-three (46.7%) of the 92 patients presented with AAC (+) test results based on the ultrasound criteria, with a higher incidence of complications (27.9%) than AAC (–) patients (0%; *P* < 0.001). The median length of symptoms (8 vs. 4 days, *P* < 0.001) and duration of antibiotic therapy (13 vs. 5 days, *P* < 0.001) were longer in the AAC (+) group.

**Conclusions:** The ultrasound-based AAC (+) group often had a worse prognosis than the AAC (–) group. Therefore, patients from the AAC (+) group should receive a follow-up ultrasound examination to detect disease progression early.

## Introduction

In recent years, the average life expectancy has been steadily increasing in many countries (1). For example, China has experienced a significant increase in life expectancy, with the older population growing at a rate of 5% per year. In fact, it is expected that there will be more than 74 million individuals above 80 years of age by 2040 (2). This patient population represents a clinical challenge as older individuals are at greater risk of presenting with an episode of acute cholecystitis (AC) due to the organ dysfunction and weakened immune system, and up to 6% of older patients experience severe AC (3). Acute acalculous cholecystitis (AAC) is a gallstone-free necrotizing inflammation of the gallbladder. Approximately 5–10% of all cases of AC were AAC, which tends to be more intricate with poor prognosis compared with acute calculous cholecystitis (ACC) (4, 5) as the comorbidities such as hypertension, coronary heart disease and diabetes in elder with AAC may anonymize the progress the disease. However, the gallbladder necrosis rate of AAC is about 40–60%, while the gallbladder perforation rate is as high as 5–15%. AAC is associated with a high mortality (30% in most studies; range 10–90% with early or late diagnosis, respectively) (6).

Given the high morbidity and mortality of gallbladder gangrene and perforation that develops from ischemia in a short period of time, early and accurate diagnosis is essential. The manifestations of AAC differ from the population of patients. In children, AAC is the most frequent form of acute cholecystitis (about 50–70%), which mainly results from infectious diseases (such as parasite, Epstein-Barr virus and hepatitis A virus infections). Thus, supportive care (analgesia, rehydration) is usually the primary treatment, together with antibiotic therapy, regular clinical evolution and sonographic monitor (7). While in adults, AAC is often secondary to severe trauma, post resuscitation, different kinds of shock, major surgery, long-term fasting, parenteral nutrition and severe infection, and open or laparoscopic cholecystectomy or percutaneous cholecystostomy are usually the primary treatment besides antibiotic therapy (8). However, in the elder, most patients have a more significant burden of comorbidities, especially for long-term bedridden patients; moreover, some severe patients cannot communicate their symptoms due to anesthetized, intubated, and/or unconscious situation. Furthermore, the unspecific symptoms and laboratory examinations also impede the diagnosis of AAC, including fever, pain in right upper quadrant, leukocytosis, and increased hepatic enzyme levels (9, 10). Susceptible factors in long-term bedridden elderly (such as long-time of enteral fasting, mostly parenteral nutrition, high rate of ventilator use) contribute to the worse prognosis by prolonging time of cholestasis and induce severe complications, which refer to gallbladder perforation, gallbladder hemorrhage, perigallbladder abscess.

Although various composite approaches are applied in clinical work to diagnose AAC, imaging technology is vital for the ultimate diagnosis of AAC. Ultrasound is strongly recommended by the Tokyo Guidelines 2018 (TG13) as the preferred imaging examination for the morphological diagnosis of AC given that it is easy-to-use, easy-to-repeat at the bedside, and provides a real-time assessment (11). Ultrasound is of greater significance in elderly patients with long-term bedridden as this group of people rely more on bedside ultrasound for follow-up, to provide timely information of disease changes. Moreover, ultrasound of the gallbladder is believed to be the most accurate method for diagnosing AAC in critically ill patients (6).

In long-term bedridden elderly patients, however, disease characteristics, comorbidities, and poor functional status augment the rates of misdiagnosis and missed diagnosis by ultrasound. In these patients, AAC often represents further progression of multiple systemic failures. To the best of our knowledge, the occurrence of AAC is rarely investigated as a medical complication in elderly long-term bedridden patients. Although such cases are frequently encountered clinically, only several case reports are available (12, 13). Moreover, no studies have specifically demonstrated the prognostic value of the current ultrasound criteria for AAC in this patient group.

Given the fact that the patient population included represents a clinical challenge as older individuals are at greater risk of presenting with an episode of acute cholecystitis (AC) due to the organ dysfunction and weakened immune system. Up to 6% of older patients experience severe AC, with little systematic information on further study of the elder with AAC. What's more, the relationship between diagnostic criteria, severity classification and treatment indications for the vulnerable subgroup is still limited. Therefore, the aim of the current study was to investigate the necessity of ultrasound in early AAC diagnosis and prognosis improvement in this specific population to improve clinical management and treatment planning.

## Materials and Methods

### Research Participants

This retrospective cohort study was performed in Chinese PLA General Hospital. The study protocol was officially approved by ethics committee of Chinese PLA General Hospital, Beijing (No. S2021-230-01, obtained on March, 2021) and conformed to the Declaration of Helsinki. According to the Ethics Committee, our study is retrospective research, therefore, patient consent statements were waived and each patient had signed the standard consent statement before ultrasound. Our study population was selected from 374 elderly bedridden patients (bed rest time ≥ 20 days, mean bedtime 92.35 ± 29.47 days) with multiple comorbidities (diabetes, cardiac disease, obstructive pulmonary diseases, and renal insufficiency) and clinical findings supportive for AC (fever, right upper quadrant abdominal pain, increased abnormal liver function indicators, and tenderness), who underwent ultrasound examinations from January 1, 2018 to May 1, 2020.

Ultrasound is used as the method for diagnosing AAC in our analysis, subsequent imaging (CT or MRI) or pathological examinations are auxiliary tools if there is still uncertainty about the diagnosis. Patients with AC were included into our study initially and patients with cholecystolithiasis/choledocholithiasis, ACC, history of cholecystectomy, malignant tumor, liver disease, and those with uncertain results of cholecystectomy were excluded out of analysis totally. The exclusion criteria included patients with hepatobiliary malignancy, concomitant acute cholangitis or common duct stones, chronic cholecystitis, and nonspecific gallbladder wall thickening associated with acute pancreatitis, hepatitis, pyelonephritis, peritonitis, ascites, hypoalbuminemia, congestive heart failure, or chronic renal failure.

The diagnosis criteria of AAC are recommended by the Tokyo Guidelines 2018 (TG13) as follows. A. Local signs of inflammation etc. (1) Murphy's sign, (2) RUQ mass/pain/tenderness. B. Systemic signs of inflammation etc. (1) Fever, (2) elevated CRP, (3) elevated WBC count. C. Imaging findings. Imaging findings characteristic of acute cholecystitis. On the premise of excluding acute hepatitis, other acute abdominal diseases, and chronic cholecystitis and no calculus found, suspected diagnosis of AAC is made on the condition of one item in A + one item in B and definite diagnosis is made on the condition of one item in A + one item in B + C (11).

Among the 374 patients, 282 patients were excluded from our analysis for above reasons, which will be described in detail later and a total of 92 patients suspected of AAC were included in the analysis which were divided into AAC (+) and AAC (–) groups according to TG13.

### Ultrasound Protocol

Ultrasound examinations were carried out with a PHILIPS CX50 portable ultrasound machine (PHILIPS Medical System, Bothell, WA, USA) equipped with a curved transducer (3–7 MHz). Three certified ultrasound technicians with more than 5 years of work experience performed the examination using a standardized ultrasound examination protocol. The ultrasound examination procedures, including standard image acquisition and measurements, were strictly recorded, and each examiner followed the same protocol to minimize the information bias between examiners. The ultrasound images stored in the PACS system were reviewed and recorded by two independent observers.

**Figure 1:**
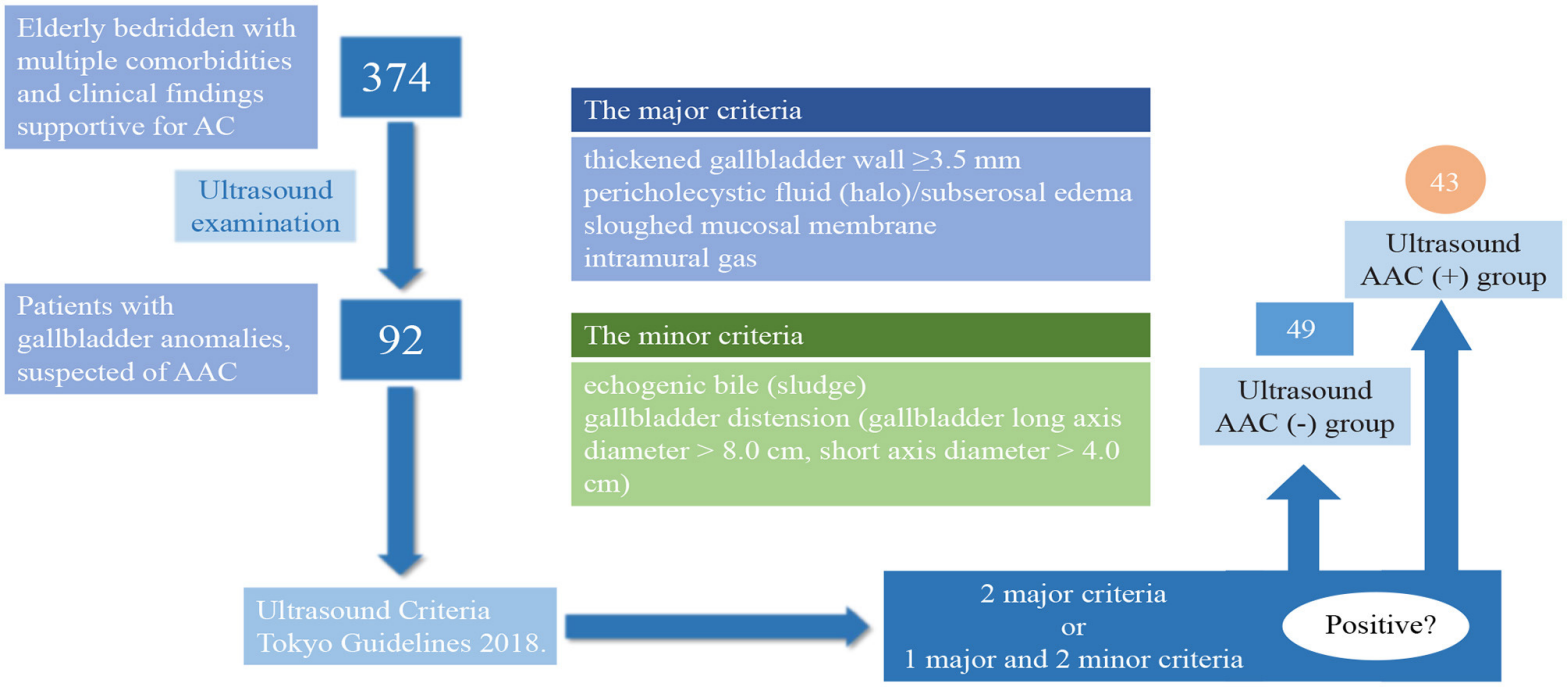
AAC ultrasonic examination criteria and a flowchart detailing the patient flow.

**Figure 2:**
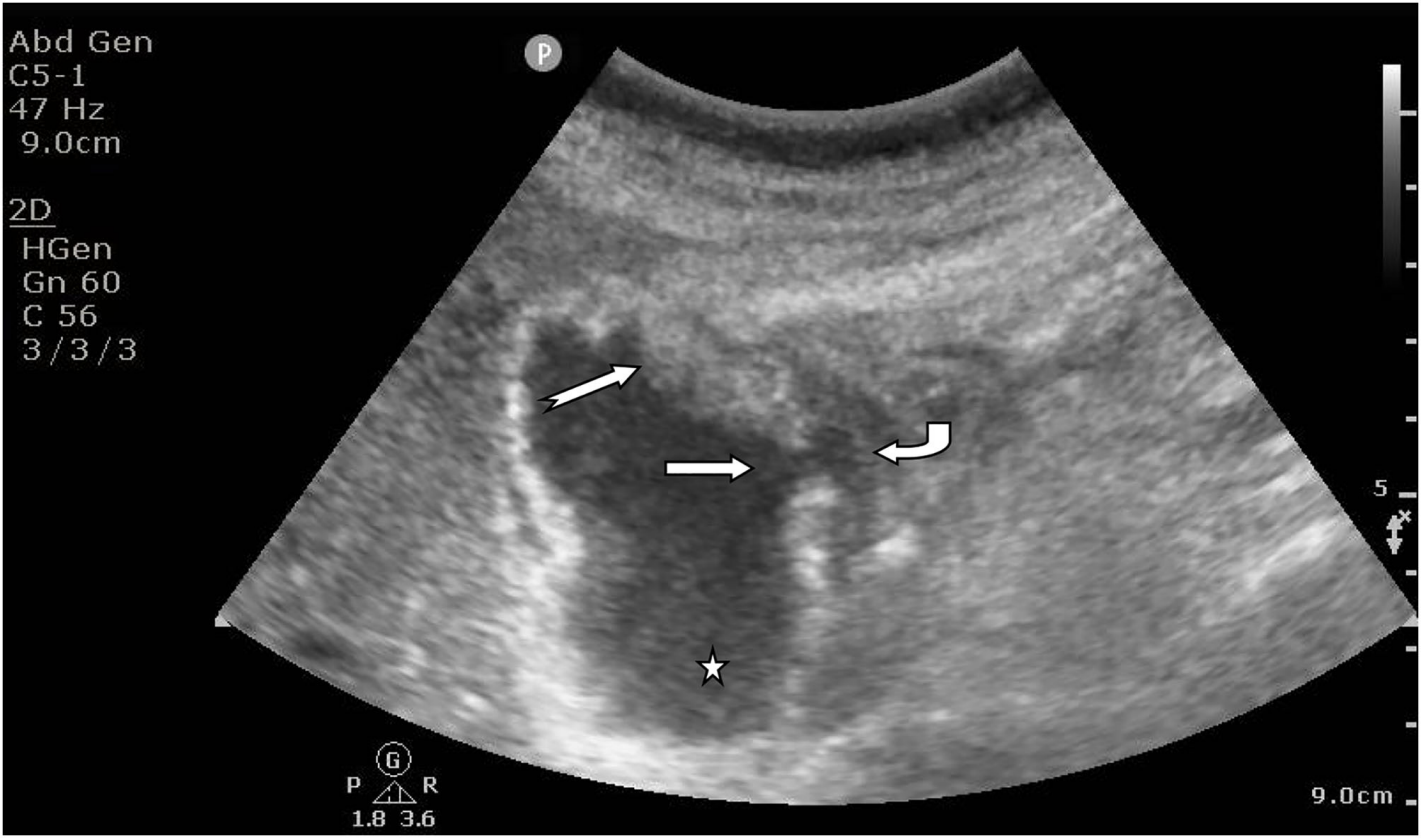
Supine examination of a male patient showed increased gallbladder volume, interrupted continuity of the gallbladder wall, perforation (straight arrows), uneven thickening of the gallbladder wall with mucosal layer abscission (swan-tail arrows), cholestasis (pentagonal star), and effusion around the gallbladder (curved arrows).

AAC ultrasonic examination criteria and a flowchart detailing the patient flow are shown in Figure 1. Patients were considered AAC (+) if at least two major criteria or one major with two minor criteria were identified from the ultrasound examination. The major criteria included: a thickened wall of the gallbladder (≥ 3.5 mm), pericholecystic fluid (halo)/ subserosal edema, a sloughed mucosal membrane, and intramural gas. The minor criteria included: echogenic bile (sludge) and gallbladder distension (gallbladder long axis diameter > 8.0 cm, short-axis diameter > 4.0 cm) (6). The typical ultrasonic manifestations of AAC are shown in Figure 2.

In addition, we reviewed the clinical course, including symptoms, complications, treatment, ultrasound imaging findings, and outcome (mortality) in all the patients. The duration of ultrasound follow-up in our study was at least 2 weeks since the initial clinical suspicion.

Our analysis brought into only complications associated with cholecystitis acquired during subsequent imaging (CT or MRI) or pathological examinations, including gallbladder perforation, gallbladder abscess, gallbladder bleeding, and cholecystitis-associated septicemia.

All the databases and files were retrospectively reviewed with approval from the local ethics committee.

### Data Analysis and Statistics

SPSS Statistics version 18.0 (IBM Corp., Armonk, N.Y., USA) was used for the statistical analysis. Categoric variables are presented as numbers and percentages; continuous variables are summarized using means ± standard deviations. The Wilcoxon rank-sum test and chi-square test or Fisher's exact test were used to compare the duration of symptoms and the complication rates between the groups, respectively. A *P* < 0.05 was considered statistically significant.

## Results

### Clinical Characteristic

Among the 374 patients, 97 with cholecystolithiasis/choledocholithiasis confirmed by imaging data, 59 with ACC, 45 with a history of cholecystectomy, 39 with liver disease that may cause symptoms of right upper abdominal pain, 30 with a malignant tumor, and 12 with uncertain results of cholecystectomy were excluded. Therefore, a total of 92 patients suspected of AAC were included in the analysis.

The clinical characteristics were shown in Table 1. Their mean age was 81.92 ± 19.68 (58–101) years, and 69.57% were men. There were nine patients (9.78%) with a history of recent major surgery, 15 (16.30%) with prolonged enteral fasting, 28 (30.43%) with parenteral nutrition, 25 (27.17%) on mechanical ventilation, and 15 (16.30%) under sedation.

**Table 1:** Patients' demographic and clinical characteristics.

	**Overall patients** ***n* = 374**	**Included patients** ***n* = 92**
Age, years, mean (SD)	84 (12)	82 (8.0)
Gender, male (%)	214 (57.22)	64 (69.57)
**Co-morbidities**, ***n*** **(%)**		
Diabetes mellitus	132 (35.29)	32 (33.70)
Cardiac disease	107 (28.61)	30 (32.61)
COPD and/or asthma	61 (16.31)	14 (15.22)
Chronic renal failure	49 (13.10)	9 (9.78)
Biliary disease history	198 (52.94)	44 (47.83)
**Clinical presentation**		
RUQ pain/ mass /tenderness	223 (59.63)	65 (70.65)
Fever	179 (47.86)	40 (43.48)
Vomiting	163 (43.58)	39 (42.39)
Jaundice	55 (14.33)	26 (28.26)
Hypotension^a^	23 (6.15)	9 (9.78)
**Laboratory data**		
WBC (10^9^/L)	12.5 (8.8, 16.2)	12.3 (8.5, 15.7)
Platelets (10^9^/L)	191 (152, 250)	194 (159, 250)
CRP (mg/L)	119.6 (98.9, 129.4)	125.3 (108.7, 130.1)
Albumin1 (g/L)	35 (27, 38)	34 (28, 39)
Bilirubin1 (μmol/L)	59 (37, 92)	67 (44, 96)
ALT (IU/L)	127(66, 251)	139 (85, 266)
AST (IU/L)	159 (76, 359)	180 (89, 370)
ALP (IU/L)	200 (127, 334)	214 (117, 340)
GGT (IU/L)	258 (127, 446)	286 (159, 523)
INR	1.15 (1.10, 1.30)	1.18 (1.10 1.30)
**Radiological investigations**		
US scan, *n* (%)	374 (100)	92 (100)
CT scan, *n* (%)	162 (43.32)	35 (38.04)
MRI scan, *n* (%)	83 (22.19)	14 (15.22)
**AC grade**		
Grade I	89 (23.80)	24 (26.09)
Grade II	178 (47.59)	43 (46.74)
Grade III	107 (28.61)	25 (27.17)

a *Hypotension was defined as systolic blood pressure of <90 mmHg.*

*COPD, Chronic obstructive pulmonary disease; RUQ, Right upper quadrant; WBC, White blood cell; CRP, C-reactive protein; ALT, Alanine aminotransferase; AST, Aspartate aminotransferase; ALP, Alkaline phosphatase; GGT, Gamma-glutamyl transferase; INR, International normalized ratio; US, Ultrasound; CT, Computed tomography; MRI, Magnetic resonance imaging; AC, Acute cholecystitis. All cases were classified into Grade I (mild), Grade II (moderate), and Grade III (severe) AC according to Tokyo 13/18 Guidelines*.

### Ultrasound Examination

Patients were divided into ultrasound AAC (+) and ultrasound AAC (–) groups. Symptom duration, complications, and gallbladder-associated mortality were compared between the two groups.

Among these 92 patients, 43 cases (46.74%) were diagnosed by ultrasound as AAC (+) and 49 (53.26%) as AAC (–). Additionally, among these 92 patients, ultrasound examinations revealed mucosal layer shedding in the gallbladder wall in 15 cases (16.30%), effusion around the gallbladder in 45 (48.91%), gallbladder distension in 55 (59.78%), thickening of the gallbladder wall in 65 (70.65%), and sludge in 80 (86.96%). No gas in the gallbladder wall was found in any of the ultrasound examinations. The ultrasound characteristics of the AAC (+) and AAC (–) patients are shown in Table 2.

**Table 2:** Summary of ultrasound imaging characteristics in acute acalculous cholecystitis (AAC) positive and negative groups.

**Characteristic**	**Total, *n***	**Positive^**a**^**	**Negative^**b**^**	**Chi square**	***P***
**Ultrasound Diagnosis**, ***n*** **(%)**					
**Wall thickness** **≥** **3.5 mm**				19.486	0.000
No	27	3 (11.11)	24 (88.89)		
Yes	65	40 (61.54)	25 (38.46)		
**Pericholecystic fluid**				11.092	0.001
No	47	14 (29.79)	33 (70.21)		
Yes	45	29 (64.44)	16 (35.56)		
**Sloughed mucosa**				20.423	0.000
No	77	28 (36.36)	49 (63.64)		
Yes	15	15 (100.00)	0 (0.00)		
**Intramural gas**					
No	92	25 (27.17)	67 (72.83)		
**Hydrops**				19.242	0.000
No	37	7 (18.92)	30 (81.08)		
Yes	55	36 (65.45)	19 (34.55)		
**Echogenic bile/sludge**				2.620	0.106
No	12	3 (25.00)	9 (75.00)		
Yes	80	40 (50.00)	40 (50.00)		
Total	92	43 (46.74)	49 (53.26)		

a *Ultrasound AAC (+) group,*

b *Ultrasound AAC (-) group*.

### Complications

Complications occurred in 12 patients from the AAC (+) group, including four cases of gallbladder perforation, three of gallbladder bleeding, three of pericholecystic abscess, and two of septicemia. The ultrasound diagnosis of the AAC (+) group was significantly related to the occurrence of complications (27.91%). In contrast, no complications occurred in the AAC (–) group (*P* = 0.005, Table 3).

**Table 3:** Summary of complications by ultrasound in acute acalculous cholecystitis (AAC) positive and negative groups.

**Complications**	**Total, *n***	**Positive^**a**^**	**Negative^**b**^**	**Chi square**	***P***
**Ultrasound Diagnosis**, ***n*** **(%)**					
**Perforation**					0.044
No	88	39 (44.32)	49 (55.68)		
Yes	4	4 (100)	0 (0.00)		
**Gallbladder hemorrhage**					0.098
No	89	40 (44.94)	49 (55.06)		
Yes	3	3 (100.00)	0 (0.00)		
**Pericholecystic abscess**					0.098
No	89	40 (44.94)	49 (55.06)		
Yes	3	3 (100.00)	0 (0.00)		
**Septicemia**					0.216
No	90	41 (45.56)	49 (54.44)		
Yes	2	2 (100.00)	0 (0.00)		
**Total**				15.726	0.000
No	80	31 (38.75)	49 (61.25)		
Yes	12	12 (100.0)	0 (0.00)		

a *Ultrasound AAC (+) group,*

b *Ultrasound AAC (-) group*.

In addition, ultrasound examinations revealed that pericholecystic effusion (11 of 45 patients, 24.44%), gallbladder distension (12 of 55 patients, 21.81%), and sludge (12 of 80 patients, 15.00%) were related to the occurrence of complications (Table 4). In the AAC (+) group, sloughed mucosa was observed in 15 cases (34.89%), although only four of these presented with complications (*P* > 0.99).

**Table 4:** Summary of patient characteristics and ultrasound features by complication status.

**Characteristic**	**No**	**Yes**	**Chi square**	***P***
**Any Complication, n (%)**				
**Wall thickness** **≥** **3.5 mm**				
No	27 (100.00)	0 (0.00)		0.016
Yes	53 (81.54)	12 (18.46)		
**Pericholecystic fluid**				
No	46 (97.87)	1 (2.13)	10.095	0.001
Yes	34 (75.56)	11 (24.44)		
**Sloughed mucosa**				
No	69 (89.61)	8 (10.39)		0.103
Yes	11 (73.33)	4 (26.67)		
**Intramural gas**				
No	87 (94.57)	5 (5.43)		
**Hydrops**				
No	37 (100.00)	0 (0.00)		0.001
Yes	43 (78.18)	12 (21.82)		
**Echogenic bile/sludge**				
No	12 (100.00)	0 (0.00)		0.354
Yes	68 (88.00)	12 (15.00)		
**Ultrasound diagnosis of AAC**				
Positive	31 (72.09)	12 (27.90)	15.726	0.000
Negative	49 (100.00)	0 (0.00)		
**Results of two imaging diagnoses**				
Both negative	29 (100.00)	0 (0.00)		0.016
Any positive	51 (80.95)	12 (20.83)		
Total	80 (86.96)	12 (13.04)		

We also analyzed and compared hospital outcomes of AAC patients who with and without complications (Table 5). There was no difference in ultrasound-guided percutaneous cholecystostomy (US-PC) between two groups (12 vs. 10; *p* = 0.961). However, 12 AAC patients with complications (100%) underwent US-PC. 4 AAC patients were treated with Cholecystectomy, which were all came from the complications group. There was no patient with complications underwent antibiotics only. The length of ICU stay and follow-up time were comparable between two groups (24.5 vs. 12.9, 33.5 vs. 19.1, *p* < 0.05). (There were two deaths in the complications group, but there was no correlation with gallbladder pathology).

**Table 5:** Treatment and outcomes of AAC patients with complications vs. without complications.

	**Total (*n* = 43)**	**Complications (*n* = 12, 27.91%)**	**Without complications** **(*n* = 31,72.09%)**	***p*-value**
**Treatment**				
US-PC	22 (68.75)	12 (100.00)	10 (32.26)	NS
Cholecystectomy^a^	4 (100.0)	4 (100.0)	0 (0.0)	<0.05
Antibiotics only	17 (30.4)	0 (0.0)	17(54.8)	<0.05
**Outcomes**				
ICU cases	10 (23.3)	6 (50.0)	6 (19.4)	NS
Length of ICU stay, days	16.5 (5.6–18.5)	24.5 (14.0–47.3)	12.9 (4.9–18.3)	<0.05
Mortality (Indirectly associated with AAC)	2 (4.7)	2 (16.7)	0 (0.0)	<0.05
Follow-up time (weeks)	21.6 (4.0–36.5)	33.5 (8.7–40.6)	19.1 (4.0–25.3)	<0.05

*US-PC, Ultrasound-guided percutaneous cholecystostomy; ICU, intensive care unit.*

a *Ultrasound and pathologic findings were congruent in these patients*.

### Follow-Up Ultrasound Examination and Treatment

In the follow-up ultrasound examinations of the 43 patients in the AAC (+) group, 15 (34.88%) still presented as AAC (+). For these 15 patients, the ultrasound follow-up frequency was two to three times in the first 2 weeks and once a week in the following weeks until the symptoms disappeared or until death (mean follow-up time: 21.63 ± 12.67 weeks, range: 4–36 weeks). Another 28 patients (65.12%) in the positive group presented with negative findings for AAC in the follow-up ultrasound examinations after treatment. Of note, during the follow-up, only one AAC (+) patient worsened; in this case, a cholecystoduodenal fistula was identified by ultrasound in time for treatment, and the patient recovered after treatment. There were two deaths in the AAC (+) group, although there was no correlation with gallbladder pathology. In the AAC (-) group, 19 (38.78%) of the 49 patients were diagnosed as AAC (+) on follow-up diagnostic tests within 1 week. Among these 19 patients, 11 cases (57.89%) were diagnosed upon ultrasound re-examination, five (26.31%) with CT, and three (15.79%) with MRI. Following treatment, all of the patients in the AAC (-) group were experienced a complete disappearance of fever and right upper quadrant pain.

Significant alleviation or resolution of cholecystitis manifestations occurred in 17 of 43 patients in the AAC (+) group and 39 of 49 in the AAC (–) group following treatment with antibiotics. Twenty-two AAC (+) patients and 10 AAC (–) patients underwent ultrasound-guided percutaneous cholecystostomy (US-PC). Only four AAC (+) patients received cholecystectomy at the initial occurrence.

The median length of symptoms (eight vs. four days, *P* < 0.001) and duration of antibiotic therapy (13 vs. 5 days, *P* < 0.001) were longer in the AAC (+) group (Table 6).

**Table 6:** Summary of clinical treatment and the duration of cholecystitis symptoms and antibiotic therapy by ultrasound follow-up.

**Items**	**Ultrasound Diagnosis**		**Total, *n***
**Treatment**	**Positive^**b**^ (*n* = 43)**	**Negative^**c**^ (*n* = 49)**	
US-PC	22 (68.75)	10 (31.25)	32
Cholecystectomy^a^	4 (100.00)	0 (0.00)	4
Antibiotics	17 (30.36)	39 (69.64)	56
**Duration time**			***P*** **value**
Length of symptoms, median (IQR^d^), days	8 (4–15)	4 (1–6)	<0.001
Antibiotic duration, median (IQR^d^), days	13 (10–17)	5 (4–8)	<0.001

*US-PC, Ultrasound-guided percutaneous cholecystostomy.*

a *Ultrasound and pathologic findings were congruent in these patients,*

b *Ultrasound AAC (+) group,*

c *Ultrasound AAC (-) group,*

d *The median interval (IQR)*.

## Discussion

It has been suggested that the changes in the gallbladder observed with AAC may be a manifestation of systemic illnesses, such as intercurrent infections, metabolic disorders, vascular problems, injuries, and malignancies (14–17). Importantly, AAC mainly occurs in patients with debilitating conditions, and the development of AAC in elderly patients has already been reported with a male predominance (18). In our study, the proportion of older men was as high as 69.57% (64/92). All 92 patients suspected of AAC presented with systemic diseases of varying severity (9.78% stayed in the intensive care unit for significant surgery, 16.30% were on prolonged enteral fasting, 30.43% on parenteral nutrition, 27.17% on mechanical ventilation, and 16.30% under sedation). AAC poses significant diagnostic challenges in this group because it is generally a secondary event that occurs in acute-developed patients with complications. Therefore, the sensitivity and specificity of clinical manifestations are reduced, and an abdominal ultrasound is requested for the first time.

In our study, the analysis was carried out using only the ultrasound criteria. The use of ultrasound in AAC has been well described in case series studies (6, 11). Although ultrasound diagnostic criteria and its diagnostic rate in the diagnosis of AAC varies among studies, diagnosing AAC based on identifying major and minor criteria is considered to be reasonably sensitive (19, 20). The role of diagnostic ultrasound in patients with AAC is twofold; namely, it is used to clarify the diagnosis in suspected cases based on clinical and laboratory results as well as to detect complications. Our results suggest that ultrasound diagnosis of AAC (+) in long-term bedridden elderly patients is predictive of a higher incidence of complications and a longer duration of clinical manifestation. The complication rate in the AAC (+) group was 27.91% in this study [compared with 0% in the AAC (–) group]. The higher incidence rate observed in our study agrees with previous studies (18–20). For example, a previous study found that among 94 patients with hematological malignancies and clinical manifestations, the AAC (+) group often presented with a higher rate of complications and mortality (20.9%) than the AAC (–) group (0%; *P* < 0.001) (21).

Although the pathogenesis of AAC is largely unknown, it is most likely related to bile stasis, necrosis, and ischemia of the gallbladder wall (22). In our study, all the AAC patients were found to have atherosclerotic vascular-associated diseases, such as hypertension, diabetes, apoplexy, or ischemic heart disease. Studies have shown that atherosclerosis and increased blood viscosity in the elderly aggravates the ischemia of the gallbladder wall (23). In particular, diffuse damage to the wall of small blood vessels, which narrows and obstructs the arterial lumen, and damage to gallbladder mucosa significantly increase the risk of AAC (16, 24–26). In fact, a study by Mungazi et al. (27) confirmed that systemic arteriosclerosis can cause a blood flow obstruction in the gallbladder wall, and that this gallbladder blood flow disorder makes patients more prone to gallbladder gangrene and gallbladder perforation. It is noteworthy that although gallbladder enlargement and cholestasis were considered secondary criteria, they were found in all 12 patients with complications in our study. This is likely caused by an excessive increase in gallbladder volume and increased pressure in the gallbladder cavity that produces gallbladder ischemia, leading to gangrene and gallbladder perforation, among other complications. Previous studies have shown that bile stasis due to slow or incomplete gallbladder contraction can alter the chemical composition of bile through increasing the level of stimulators, such as lysophosphatidylcholine, which predisposes the patient to mucosal injury (28). Increased intraluminal pressure resulted from bile stasis is believed to compromise gallbladder perfusion pressure, which may get exacerbated by vasoactive drugs and hypotension in critically ill patients (28, 29). Among bedridden elder patients, risk factors for gallbladder dysmotility and bile stasis are common, such as mechanical ventilation, fasting, total parenteral nutrition, and continuous enteral feeding. Inflammation of the gallbladder wall is further aggravated in these patients, and edema of the gallbladder mucosa occurs, which affects the gallbladder vein reflux and increases the risk of gallbladder perforation (17, 30). As there is no consensus and guidelines on the management of senile cholecystitis at home and abroad. Treatment is generally based on past experience, diagnostic laboratory, clinical, and imaging examinations. Operations are not adopted in our study due to many high-risk factors such as old age, bedridden and comorbidities. Percutaneous transhepatic gallbladder drainage or conservative treatment are applied to patients to relieve clinical manifestations according to patient conditions.

In this study, ultrasound monitoring of the gallbladder contributed to the diagnosis of AAC. In addition, ultrasound examination was instrumental in monitoring abnormalities as well as for efficacy evaluation in elderly long-term bedridden patients. We considered these two aspects in our study because the use of ultrasound is well worth concerns to broaden therapy options and decrease complications in AAC. An ultrasound follow-up in highly suspicious cases facilitates early diagnosis and prompts treatment, reducing complications and mortality. In this study, 38.78% of patients in the AAC (–) group were diagnosed as AAC (+) during the first week of ultrasound or CT follow-up review, indicating the rapidly progressing nature of AAC. Nevertheless, these patients often presented with early or mild AAC manifestations with fewer complications and a shorter disease course, leading to a negative diagnosis during the initial ultrasound examination. On the other hand, the lower complication rate and shorter duration of symptoms may also attribute to early diagnosis and treatment in this group. This is in line with a study by Thampy et al. (21), which found that 41% of the patients in the AAC (–) group had positive results for AAC within 1 week of the follow-up ultrasound. Due to the rapid progression of AAC, the role of ultrasound follow-up has been emphasized for the early detection of progressed AAC as well as to prevent misdiagnoses and missed diagnoses (19). Ultrasound can also be used as an adjunct treatment in AAC patients. In our study, only four of the 92 patients underwent a cholecystectomy, which is not surprising considering that elderly patients are often poor candidates for surgery because of concomitant medical problems. Thirty-two cases [22 AAC (+) and 10 AAC (–)] received percutaneous transhepatic gallbladder drainage (PTGBD) for AC in our study. Importantly, ultrasound could be used to guide the PTGBD procedure, increasing its safety and efficacy, or to constantly monitor its therapeutic efficacy.

Our study is not without limitations. First, as a retrospective study, patient randomization was not possible. Moreover, due to the relatively small patient sample size, it was not possible to perform a subgroup analysis, and conclusions on the relationship between the laboratory test results and imaging examinations in patients with gallbladder abnormalities could not be made. Second, most of the patients (56 of 92) had no pathologic confirmation. This is mainly because the patients in the acute stage were critically ill, and all of them were older patients who typically do not tolerate a cholecystectomy well. Finally, since we aimed to assess the prognostic importance of ultrasound, the ultrasound results of the gallbladder after treatment were not well assessed. In the future, more prospective studies are required to further investigate these unsolved issues.

Despite the above limitations, ultrasound is recommended as a pivotal diagnostic technique for the early discovery and treatment of gallbladder diseases in long-term bedridden elderly patients. The appropriate application of ultrasound may be instructive in determining the most appropriate and effective therapeutic schedule for individual patients.

## Data Availability Statement

The raw data supporting the conclusions of this article will be made available by the authors, without undue reservation.

## Ethics Statement

The studies involving human participants were reviewed and approved by Ethics Committee of Chinese PLA General Hospital, Beijing. Written informed consent for participation was not required for this study in accordance with the national legislation and the institutional requirements.

## Author Contributions

YL, MY, GX, and JL: concept and design and supervision. QL, LS, CC, YQ, and ZY: acquisition, analysis, or interpretation of data and drafting of the manuscript. QL, LS, and CC: statistical analysis. MY, GX, and JL: obtained funding. The corresponding author attests that all listed authors meet authorship criteria. All authors critical revision of the manuscript for important intellectual content and read and approved the final draft of the manuscript.

## Funding

This study was supported and funded by National Key Research and Development Program (2018YFC2002400) and National Key Research and Development Program (2020YFC2002706).

## Conflict of Interest

The authors declare that the research was conducted in the absence of any commercial or financial relationships that could be construed as a potential conflict of interest.

## Publisher's Note

All claims expressed in this article are solely those of the authors and do not necessarily represent those of their affiliated organizations, or those of the publisher, the editors and the reviewers. Any product that may be evaluated in this article, or claim that may be made by its manufacturer, is not guaranteed or endorsed by the publisher.

## References

[1] Liou L Joe W Kumar A Subramanian SV. Inequalities in life expectancy: An analysis of 201 countries, 1950-2015. Social Sci Med. (2020) 253:112964. 10.1016/j.socscimed.2020.112964 32247943

[2] Aging population: China's development trend and policy options (2020). China Development Research Foundation. https://www.cdrf.org.cn (accessed June 11, 2020).

[3] Lee SW Yang SS Chang CS Yeh HJ. Impact of the Tokyo guidelines on the management of patients with acute calculous cholecystitis. J Gastroenterol Hepatol. (2009) 24:1857–61. 10.1111/j.1440-1746.2009.05923.x 19686411

[4] Kimura Y Takada T Kawarada Y Nimura Y Hirata K Sekimoto M. Definitions, pathophysiology, and epidemiology of acute cholangitis and cholecystitis: Tokyo Guidelines. J Hepato-Biliary-Pancreatic Surgery. (2007) 14:15–26. 10.1007/s00534-006-1152-y 17252293 PMC2784509

[5] McChesney JA Northup PG Bickston SJ. Acute acalculous cholecystitis associated with systemic sepsis and visceral arterial hypoperfusion: a case series and review of pathophysiology. Digestive Diss Sci. (2003) 48:1960–7. 10.1023/a:1026118320460 14627341

[6] Huffman JL Schenker S. Acute acalculous cholecystitis: a review. Clin Gastroenterol Hepatol. (2010) 8:15–22. 10.1016/j.cgh.2009.08.034 19747982

[7] Poddighe D Sazonov V. Acute acalculous cholecystitis in children. World J Gastroenterol. (2018) 24:4870–9. 10.3748/wjg.v24.i43.4870 30487697 PMC6250923

[8] Treinen C Lomelin D Krause C Goede M Oleynikov D. Acute acalculous cholecystitis in the critically ill: risk factors and surgical strategies. Langenbeck's Archives of Surgery. (2015) 400:421–7. 10.1007/s00423-014-1267-6 25539703

[9] Owen CC Jain R. Acute acalculous cholecystitis. Curr Treatment Options Gastroenterol. (2005) 8:99–104. 10.1007/s11938-005-0001-4 15769430

[10] Trowbridge RL Rutkowski NK Shojania KG. Does this patient have acute cholecystitis? JAMA. (2003) 289:80–6. 10.1001/jama.289.1.80 12503981

[11] Yokoe M Hata J Takada T Strasberg SM Asbun HJ Wakabayashi G. Tokyo Guidelines 2018: diagnostic criteria and severity grading of acute cholecystitis (with videos). J Hepato-Biliary-Pancreatic Sci. (2018) 25:41–54. 10.1002/jhbp.515 29032636

[12] Demirkan A Tanriverdi AK Çetinkaya A Polat O Günalp M. The effect of leucocytosis, gender difference, and ultrasound in the diagnosis of acute cholecystitis in the elderly population. Emergency Med Int. (2019) 2019:6428340. 10.1155/2019/6428340 31065386 PMC6466953

[13] Choo SK Park HJ Oh HK Kang YK Kim Y. Acute cholecystitis in elderly patients after hip fracture: Incidence and epidemiology. Geriatrics & Gerontol Int. (2016) 16:380–3. 10.1111/ggi.12483 25810136

[14] Healy DG Veerasingam D O'Connell PR Hurley J. Acute acalculous cholecystitis following coronary artery bypass surgery. Irish J Med Sci. (2004) 173:160–1. 10.1007/bf03167932 15693387

[15] Gu MG Kim TN Song J Nam YJ Lee JY Park JS. Risk factors and therapeutic outcomes of acute acalculous cholecystitis. Digestion. (2014) 90:75–80. 10.1159/000362444 25196261

[16] Fagan SP Awad SS Rahwan K Hira K Aoki N Itani KM. Prognostic factors for the development of gangrenous cholecystitis. Am J Surg. (2003) 186:481–5. 10.1016/j.amjsurg.2003.08.001 14599611

[17] Laurila JJ Ala-Kokko TI Laurila PA Saarnio J Koivukangas V Syrjälä H. Histopathology of acute acalculous cholecystitis in critically ill patients. Histopathol. (2005) 47:485–92. 10.1111/j.1365-2559.2005.02238.x 16241996

[18] Ryu JK Ryu KH Kim KH. Clinical features of acute acalculous cholecystitis. J Clin Gastroenterol. (2003) 36:166–9. 10.1097/00004836-200302000-00015 12544202

[19] Myrianthefs P Evodia E Vlachou I Petrocheilou G Gavala A Pappa M. Is routine ultrasound examination of the gallbladder justified in critical care patients? Crit Care Res Pract. (2012) 2012:565617. 10.1155/2012/565617 22649716 PMC3357634

[20] Mariat G Mahul P. Prév t N, De Filippis JP, Cuilleron M, Dubois F, et al. Contribution of ultrasonography and cholescintigraphy to the diagnosis of acute acalculous cholecystitis in intensive care unit patients. Intensive Care Med. (2000) 26:1658–63. 10.1007/s001340000684 11193273

[21] Thampy R Khan A Zaki IH Wei W Korivi BR Staerkel G. Acute acalculous cholecystitis in hospitalized patients with hematologic malignancies and prognostic importance of gallbladder ultrasound findings. J Ultrasound Med. (2019) 38:51–61. 10.1002/jum.14660 29708270 PMC6207468

[22] Taoka H. Experimental study on the pathogenesis of acute acalculous cholecystitis, with special reference to the roles of microcirculatory disturbances, free radicals and membrane-bound phospholipase A2. Gastroenterol JPN. (1991) 26:633–44. 10.1007/bf02781681 1752395

[23] Nikfarjam M Manya K Fink MA Hadj AK Muralidharan V Starkey G. Outcomes of patients with histologically proven acute acalculous cholecystitis. ANZ J Surg. (2012) 82:918–22. 10.1111/j.1445-2197.2012.06202.x 22943584

[24] Aydin C Altaca G Berber I Tekin K Kara M Titiz I. Prognostic parameters for the prediction of acute gangrenous cholecystitis. J Hepatobiliary Pancreat Surg. (2006) 13:155–9. 10.1007/s00534-005-1042-8 16547678

[25] Kiziltan ME Gunduz A Kiziltan G Akalin MA Uzun N. Peripheral neuropathy in patients with diabetic foot ulcers: clinical and nerve conduction study. J Neurol Sci. (2007) 258:75–9. 10.1016/j.jns.2007.02.028 17399742

[26] Theodorou P Maurer CA Spanholtz TA Phan TQ Amini P Perbix W. Acalculous cholecystitis in severely burned patients: incidence and predisposing factors. Burns. (2009) 35:405–11. 10.1016/j.burns.2008.08.003 18951710

[27] Mungazi SG Mungani H. Acalculous cholecystitis: three case reports. East and Central African Journal of Surgery. (2016) 21:109–12. 10.4314/ecajs.v21i3.17

[28] Barie PS Eachempati SR. Acute acalculous cholecystitis. Gastroenterol Clinics of North America. (2010) 39:343–57. 10.1016/j.gtc.2010.02.012 20478490

[29] Orlando R. 3rd, Gleason E, Drezner AD. Acute acalculous cholecystitis in the critically ill patient. Am J Surg. (1983) 145:472–6. 10.1016/0002-9610(83)90042-9 6188383

[30] Jansen S Doerner J Macher-Heidrich S Zirngibl H Ambe PC. Outcome of acute perforated cholecystitis: a register study of over 5000 cases from a quality control database in Germany. Surg Endosc. (2017) 31:1896–900. 10.1007/s00464-016-5190-5 27553799

